# Relationship Between Cognitive Disorder and First-Line Targeted Therapy for Oncogene Driver–Positive Patients With Non–Small Cell Lung Cancer: Prospective Cohort Study

**DOI:** 10.2196/59647

**Published:** 2025-09-18

**Authors:** Wenjun Chen, Xueyang Hu, Senbang Yao, Ziran Bi, Maoxi Chen, Huaidong Cheng

**Affiliations:** 1 Department of Oncology Phase I Clinical Center Anhui Chest Hospital Hefei China; 2 Department of Medical Oncology The First Affiliated Hospital of Anhui Medical University Hefei China; 3 Department of Medical Oncology The Second Affiliated Hospital of Anhui Medical University Hefei China; 4 Department of Oncology The Second Affiliated Hospital of Anhui Medical University Hefei, Anhui China; 5 Department of Oncology Shenzhen Clinical Medical School of Southern Medical University Shenzhen China

**Keywords:** cognitive disorder, targeted therapy, non–small cell lung cancer, quality of life, C-reactive protein

## Abstract

**Background:**

Previous studies have found and confirmed a correlation between cognitive disorder and chemotherapy. As genetic testing becomes more routine in clinical practice, targeted therapies are increasingly gaining prominence. The relationship between targeted treatment and cognitive function is not yet clear. This study aimed to investigate the correlation between cognitive disorder and targeted treatment by evaluating the changes in cognitive function before and after targeted therapy.

**Objective:**

This study aims to explore whether targeted therapy affects cognitive function in patients with advanced lung cancer and to explore the association between cognitive function, the inflammatory biomarker C-reactive protein, and psychological stress.

**Methods:**

From the screened cohort of 150 patients with advanced non–small cell lung cancer (NSCLC) with gene mutations, 87 (58%) were rigorously selected for the study. The evaluation instruments used were the Mini-Mental State Examination scale, the Distress Thermometer, and the European Organisation for Research and Treatment of Cancer Quality of Life Questionnaire Core 30 for assessing quality of life.

**Results:**

A significantly lower progression-free survival (PFS) was observed in the group of patients surviving advanced NSCLC with cognitive disorder under targeted therapy in contrast to survivors in the group with no cognitive disorder (hazard ratio=0.347, 95% CI 0.209-0.578; *P*<.001). Furthermore, the objective response rate and disease control rate for the group with cognitive disorder were noted to be 37.8% and 86.7%, respectively, contrastingly lower than those in the group with no cognitive disorder, recorded at 78.6% and 97.6%, respectively. Significant variances were also noted in the Mini-Mental State Examination scores between patients with and without cognitive disorder both before and after targeted therapy (*P*<.001 in both cases), with a decreasing trend observed in both groups after targeted therapy. Noteworthy differences were found in quality of life scores both before and after targeted therapy (*P*<.001 in both cases). In addition, notable disparities were apparent in C-reactive protein levels among the 2 groups before and after treatment (*P*=.03 and *P*=.048 for each time point, respectively), with an upward trend observed in both groups after targeted therapy. The multivariate Cox regression analysis demonstrated that cognitive function is an independent risk factor for PFS in patients with NSCLC receiving targeted therapy.

**Conclusions:**

Cognitive disorder may lead to lower quality of life scores and shorter PFS in patients undergoing targeted therapy. Early screening and intervention for such patients could effectively improve clinical outcomes and quality of life.

## Introduction

### Background

Lung cancer has become the leading cause of death worldwide, with non–small cell lung cancer (NSCLC) accounting for 85% of all cases [1]. NSCLC has various types, such as adenocarcinoma, squamous cell carcinoma, large cell carcinoma, and sarcomatoid carcinoma, with adenocarcinoma being predominant [2]. Surgery remains the definitive treatment for early-stage lung cancer; however, in clinical practice, most cases are diagnosed at an advanced stage. Platinum-based chemotherapy stands as the first-line treatment for NSCLC, yielding a median overall survival of 9.1 months and a median progression-free survival (PFS) period of 5 to 6 months [3]. The survival rate 1 year after chemotherapy is 37% [4].

Notably, the emergence of targeted therapy has improved the overall survival and PFS, as well as the quality of life (QoL), for some patients with advanced NSCLC. This population harbors sensitive gene mutations and has been recommended specific targeted therapies [5]. Epidermal growth factor receptor (EGFR) gene mutations, particularly those occurring at positions 18 to 21, are the most common, with those at positions 19 to 21 constituting over 90% of all EGFR mutations [6]. EGFR tyrosine kinase inhibitors, with their proven effectiveness, low toxicity, good tolerance, and ease of administration, are commonly used for advanced NSCLC. They function by entering the cells’ tyrosine kinase catalytic domain, blocking adenosine triphosphate binding, and inhibiting the EGFR signaling pathway, which in turn promotes tumor cell apoptosis; hampers tumor cell proliferation; and, ultimately, impedes tumor growth, thereby prolonging patient survival [7].

Classic studies such as IPASS (Iressa Pan-Asia Study) [8], First-SIGNAL (first-line single-agent Iressa versus gemcitabine and cisplatin trial) [9], WJTOG 3405 [10], EURTAC (erlotinib versus standard chemotherapy as first-line treatment for European patients with advanced EGFR mutation–positive NSCLC) [11], NEJ002 [12], OPTIMAL [13], and LUX-Lung 6 [14] have confirmed the improved PFS, overall survival, and objective response rate (ORR) in EGFR mutation–positive patients treated with tyrosine kinase inhibitors compared to the group treated with chemotherapy. Anaplastic lymphoma kinase (ALK) fusion gene mutations, common in NSCLC, are detected in 1.4% to 11.6% of cases. Among East Asians, the ALK fusion gene positivity rate is approximately 4.1% [15]. Currently, first-generation ALK inhibitors such as crizotinib [16] and second-generation ALK inhibitors such as alectinib [17] are commonly used for ALK fusion gene–positive patients, whereas the third-generation ALK inhibitor lorlatinib [18] was approved by the China National Medical Products Administration in April 2022 but is still less commonly used in clinical practice. ALK inhibitors have shown better efficacy than chemotherapy, resulting in prolonged PFS and improved ORRs [19].

### Objectives

In previous studies exploring the relationship between cognitive function and treatment, we have found that the cognitive function of patients with cancer is associated with chemotherapy and radiotherapy. For example, research by Simó et al [20] on cognitive function in patients with lung cancer has revealed declines in visuospatial ability and verbal fluency after chemotherapy, along with structural changes in gray and white matter in bilateral paralimbic integration areas. Radiotherapy can damage surrounding normal brain tissue during treatment, leading to cognitive dysfunction, especially when combined with chemotherapy [21]. This has been confirmed in studies on small cell lung cancer, where prophylactic cranial irradiation was associated with an increased risk of neurocognitive decline [22]. However, this presents a therapeutic dilemma. To avoid cognitive impairment, techniques such as hippocampal avoidance have been used during radiotherapy [23]. With the increasing prominence of targeted therapy for advanced NSCLC, its therapeutic efficacy has become a key focus of attention. Previous research has shown that the effectiveness of targeted therapy is influenced by the type of gene mutation [24], tumor type [25], presence of brain metastasis [26,27], and other factors. The relationship between targeted therapy efficacy and cognitive function remains unclear, warranting further investigation. In our study, we selected psychological distress, QoL, and C-reactive protein (CRP) as key variables based on previous research evidence. Both psychological distress and QoL are established critical factors influencing cognitive function [28-30]. Meanwhile, the inflammatory biomarker CRP plays a pivotal role in predicting and mediating cognitive function alterations [31,32]. This tripartite approach aligns with the biopsychosocial model, allowing for integrated analysis of psychological, functional, and biological pathways in cancer-related cognitive impairment.

## Methods

### Overview

This prospective cohort study explored the impact of changes in cognitive function during targeted therapy on therapeutic effect and the relationship with QoL, inflammatory markers, and psychological distress. The recruitment period for this study was from May 1, 2020, to December 31, 2023. We screened patients who were pathologically diagnosed with NSCLC and selected for analysis those with concomitant EGFR and ALK gene mutations who had not received any antitumor therapy. A total of 87 cases met the inclusion criteria. During the baseline period, we collected basic information; clinical data; and scores on the European Organisation for Research and Treatment of Cancer Quality of Life Questionnaire Core 30 (EORTC QLQ-C30), the Distress Thermometer (DT), and Mini-Mental State Examination (MMSE) scales from all participants. The MMSE is a well-validated and globally recognized tool for screening cognitive impairment widely used in both clinical and research settings due to its strong operational feasibility. Its reliability and validity have been extensively documented across diverse populations, including patients with cancer, assessing key cognitive domains affected by cancer treatments, such as orientation (time and place), memory (immediate and delayed recall), attention (serial subtraction and spelling), language abilities (naming and repetition), and visuospatial skills (figure copying) [33-35]. Consistent with previous research reports, an MMSE score of 26 or below is established as the cutoff threshold for identifying functional impairment; hence, the cutoff value was set at 26 for our study [36-38]. At 8 weeks of treatment, or when the tumor had progressed, the QoL questionnaire (EORTC QLQ-C30), DT, and MMSE scales were administered again. Informed consent was obtained by a professional oncologist.

### Participants

The inclusion criteria for this study were as follows: (1) patients with a confirmed initial pathological diagnosis of lung adenocarcinoma, with genetic testing (histological specimen) indicating EGFR or ALK mutations; (2) patients who had not received any previous treatment and were eligible for first-line targeted therapy, with targeted therapy dosage and duration in line with National Comprehensive Cancer Network guidelines, and an expected survival period of >6 months; and (3) patients with no history of mental or neurological diseases, able to communicate normally, without hearing or language impairments, and with a Karnofsky Performance Scale score of ≥80. The exclusion criteria for this study were as follows: (1) diagnosis of a mental illness (such as depression, anxiety, or dementia) and (2) severely debilitating diseases unrelated to the tumor that significantly impacted the patient’s QoL (such as heart failure, stroke, or disabilities).

### Procedure

**Figure 1:**
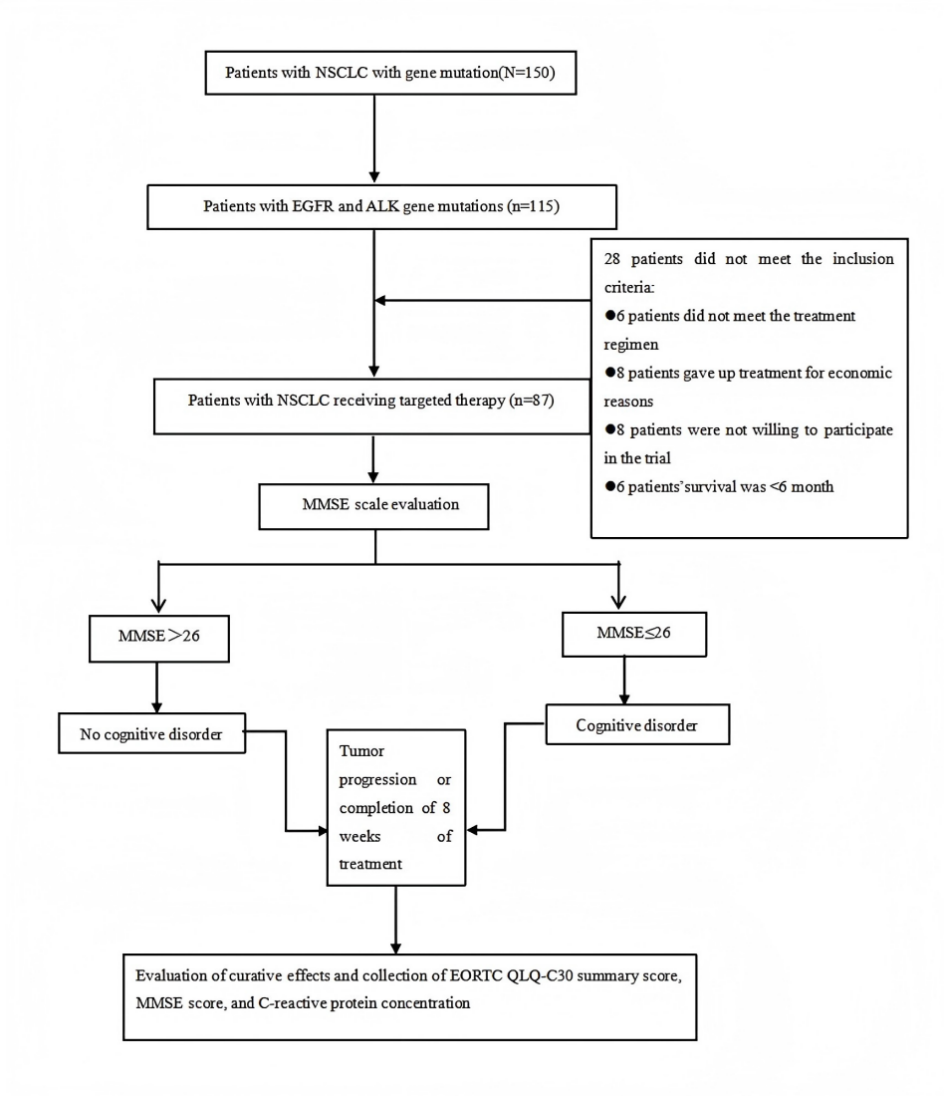
Study design flowchart. ALK: anaplastic lymphoma kinase; EGFR: epidermal growth factor receptor; EORTC QLQ-C30: European Organisation for Research and Treatment of Cancer Quality of Life Questionnaire Core 30; MMSE: Mini-Mental State Examination; NSCLC: non–small cell lung cancer.

This study was conducted at the Anhui Provincial Chest Hospital tracking patients who were diagnosed with lung cancer for the first time, and we screened 150 cases of patients with lung adenocarcinoma and gene mutations for evaluation, of whom 115 (76.7%) were detected as having mutations in EGFR and ALK genes. Informed consent was obtained through personal communication with the patients and their families, and ultimately, 87 cases participated in this study. Professional oncologists evaluated the patients; collected clinical data; and conducted assessments using the EORTC QLQ-C30, DT, and MMSE. The aforementioned 3 assessments were administered before the first treatment and after 8 weeks of targeted therapy or when the disease progressed, and the therapeutic effect was evaluated with respect to solid tumors (Response Evaluation Criteria in Solid Tumors). During the COVID-19 pandemic, patients who had difficulty visiting the hospital completed the questionnaire via video communication, and all data collected via the questionnaire were completed by professionally trained oncologists. Figure 1 shows the study design flowchart.

### Evaluation Measures

#### Gene Testing

All participants underwent histology biopsy, including lung puncture, bronchoscopy biopsy, lymph node biopsy, and surgical resection. The pathology was diagnosed by the pathology expert of the Anhui Provincial Chest Hospital pathology department, and the diagnosis was carried out by 2 individuals for diagnostic reports. The gene testing method used was high-throughput sequencing (next-generation sequencing), and the detection scope covered 139 lung cancer–related genes based on Chinese big data and the authoritative The Cancer Genome Atlas database, comprehensively covering hot spot mutations related to lung cancer (mutation, insertion, deletion, fusion, or copy number variation) and covering lung cancer therapeutic targets recommended by the National Comprehensive Cancer Network guidelines.

#### Cognitive Function Assessment

The MMSE was used for cognitive function assessment. The assessment using the MMSE questionnaire was conducted by trained professionals and consisted of 30 items covering 6 aspects. The total score out of 30 was recorded, with a score of 26 or below indicating cognitive disorder and a score above 26 indicating no cognitive disorder. The assessment was conducted twice after diagnosis, with the first assessment conducted before the first targeted therapy (time 1). The second assessment (time 2) was conducted at 8 weeks of treatment or upon tumor progression.

Important considerations included (1) ensuring that both tests were conducted in the same environment as much as possible, (2) establishing good communication, and (3) ensuring that each test item was only allowed to be attempted once (the time taken for each item may be appropriately extended to avoid the anxiety and resistance of the patient).

#### Evaluation of Targeted Therapy Efficacy

Each participant had measurable target lesions, and the Response Evaluation Criteria in Solid Tumors were used to evaluate the efficacy of targeted therapy. The efficacy assessment was divided into 4 categories: complete response, partial response, stable disease, and progressive disease. The efficacy assessments were reviewed and evaluated by 2 oncology experts.

#### Assessment Method for Psychological Distress

The DT is a tool used to assess psychological distress. It is globally applicable and convenient for clinical applications and self-assessment. It uses an 11-point visual analog scale ranging from 0 (no distress) to 10 (extreme distress) to visually represent the level of distress of the individual being tested. The DT often uses a cutoff point of 4 to distinguish between the presence and absence of distress, with higher scores indicating greater distress. When assessing the source of distress, a questionnaire consisting of 39 items is used, covering practical, familial, physical, emotional, and spiritual or religious issues. The assessments were carried out before the initiation of targeted therapy and after 8 weeks of targeted therapy or upon disease progression after targeted therapy.

#### EORTC QLQ-C30 Scale

The assessment of QoL used the EORTC QLQ-C30. The EORTC QLQ-C30 is summarized into 5 functional scales, 3 symptom scales, a global QoL scale, and 6 single items (decreased appetite, diarrhea, dyspnea, constipation, insomnia, and economic impact). The questions are divided into 4 levels, with the scores recorded as 1 to 4 points, but the global QoL scale questions adopt a 7-level answer format, with the ends being “very poor” and “excellent” and the score recorded as 1 to 7 points. The calculation method is to add the number of entries included in a specific scale or domain and divide by the number of included entries to obtain the score of each field. To make the scores of each field comparable, the scores can be linearly transformed into standardized scores within the range of 0 to 100. The comprehensive score of the EORTC QLQ-C30 is calculated as the average of the combination of 13 EORTC QLQ-C30 scale and item scores (excluding the global QoL scale and financial impact item).

#### Method for the Detection of CRP

We used a highly sensitive CRP testing kit that adopts the latex-enhanced immunoturbidimetric method to detect the content of CRP in human samples, which is used clinically as a nonspecific inflammation marker and for assessing cardiovascular disease risk. The sample requirement is fresh human ethylenediaminetetraacetic acid anticoagulated whole blood. Contaminated samples cannot be used.

The testing principle is that the reagent and sample are mixed, and the antibody-marked latex microparticles in the reagent and the CRP in the sample produce an agglutination reaction, leading to an increase in the turbidity of the solution. The concentration of CRP in the sample is obtained through turbidity analysis. This concentration is further converted into the concentration of CRP in the serum or plasma based on the proportion of blood cell volume in the sample.

### Statistical Analysis

All statistical analyses in this study were conducted using SPSS (version 24; IBM Corp). Continuous variables were tested for normal distribution using the Kolmogorov-Smirnov test. Nonparametric tests were used for variables that did not conform to the normal distribution. Baseline differences in demographic and medical characteristics between groups were independently analyzed using 2-tailed *t* test or chi-square analysis. The QoL scores in the group with cognitive disorder and the group with no cognitive disorder were compared using 2 independent-sample *t* tests. Nonparametric tests were used to compare cognitive function changes and CRP concentration changes between the 2 groups, and linear regression was used to assess the correlation between MMSE scores and CRP concentration, QoL, and psychological distress, as well as the correlation between psychological distress and CRP concentration. We used Cox regression analysis to study the impact of relevant factors on the survival rates of the 2 groups and analyze the risk factors in all participants. All statistical tests were 2-tailed, and the significance level was set at *P*<.05. Results were expressed as means and SDs.

### Ethical Considerations

This study was approved by the Biomedical Ethics Committee of Anhui Medical University (20200428). This study was conducted in accordance with the principles outlined in the Declaration of Helsinki. All patients were informed that the collected data would be used for research purposes and were assured that the study involved no invasive examinations or investigational drugs. Written informed consent was obtained from all participating patients. Participants retained the right to withdraw from the trial at any time without penalty or retaliation, and researchers were prohibited from influencing patients' decisions. Non-anonymized study data are accessible exclusively to investigators, with all data undergoing de-identification procedures prior to processing and analysis

## Results

### Demographic and Clinical Characteristics

As shown in Figure 1, there was a total of 150 patients with confirmed NSCLC and gene mutations during the screening phase of this study. Ultimately, 58% (87/150) of the patients met the inclusion criteria. The patients were divided into 2 groups, a group with no cognitive disorder (42/87, 48%) and a group with cognitive disorder (45/87, 52%), based on the MMSE assessment scores.

Table 1 shows the demographic and clinical characteristics of the study population. There were no significant differences between the 2 groups, including in age (t_79_=0.543; *P*=.59), sex (*χ*^2^_1_=0.03, *P*=.86), educational level (*χ*^2^_3_=1.2; *P*=.76), tumor stage (*χ*^2^_2_=1.2; *P*=.55), presence of distant metastasis (*χ*^2^_1_=0.2; *P*=.63), distribution of distant metastatic sites (*χ*^2^_2_=1.1; *P*=.57), targeted therapy drugs (*χ*^2^_4_=4.0; *P*=.41), and Karnofsky Performance Scale (*χ*^2^_1_=0.3; *P*=.59).

**Table 1:** Demographic characteristics and clinical data of patients with non–small cell lung cancer with genetic mutation in the groups with and without cognitive disorder (CD).

Characteristic		No CD group (n=42)	CD group (n=45)	*t* test (*df*)		Chi-square (*df*)		*P* value
Age (y), mean (SD)		58.52 (8.54)	59.73 (12.05)	0.543 (79)		—^a^		.59
**Sex, n (%)**					—		0.03 (1)	.86
	Male	16 (38)	18 (40)					
	Female	26 (62)	27 (60)					
**Educational level, n (%)**					—		1.2 (3)	.76
	Illiteracy	15 (36)	14 (31)					
	Primary school	14 (33)	14 (31)					
	Middle school	11 (26)	16 (36)					
	University	2 (5)	1 (2)					
**Tumor stage, n (%)**					—		1.2 (2)	.55
	3	7 (17)	4 (9)					
	4A	14 (33)	16 (36)					
	4B	21 (50)	25 (56)					
**Presence of distant metastasis, n (%)**					—		0.2 (1)	.63
	Yes	24 (57)	28 (62)					
	No	18 (43)	17 (38)					
**Distribution of distant metastatic sites, n (%)**					—		1.1 (2)	.57
	Bone	22 (52)	19 (42)					
	Brain	5 (12)	6 (13)					
	Liver	4 (10)	7 (16)					
**Targeted therapy drugs, n (%)**					—		4.0 (4)	.41
	First-generation EGFR^b^ TKIs^c^	24 (57)	28 (62)					
	Second-generation EGFR TKIs	5 (12)	5 (11)					
	Third-generation EGFR TKIs	10 (24)	3 (7)					
	First-generation ALK^d^ TKIs	3 (7)	3 (7)					
	Second-generation ALK TKIs	3 (7)	3 (7)					
**KPS^e^, n (%)**					—		0.3 (1)	.59
	80	20 (48)	24 (53)					
	90	22 (52)	21 (47)					

^a^Not applicable.

^b^EGFR: epidermal growth factor receptor.

^c^TKI: tyrosine kinase inhibitor.

^d^ALK: anaplastic lymphoma kinase.

^e^KPS: Karnofsky Performance Scale.

### Therapeutic Effect Between the Groups With and Without Cognitive Disorder

Table 2 shows the differences in the efficacy of targeted therapy between the groups with and without cognitive disorder. Neither group had participants reach complete response. In the group with cognitive disorder, the efficacies were partial response for 38% (17/45) of the patients, stable disease for 49% (22/45) of the patients, and progressive disease for 13% (6/45) of the patients, resulting in an ORR of 37.8% and a disease control rate (DCR) of 86.7%. In the group with no cognitive disorder, the efficacies were partial response for 79% (33/42) of the patients, stable disease for 19% (8/42) of the patients, and progressive disease for 2% (1/42) of the patients, resulting in an ORR of 78.6% and a DCR of 97.6%. There was a significant difference in the efficacies of targeted therapy between the 2 groups (*χ*^2^_2_=15.1; *P*=<.001).

**Table 2:** Comparison of the efficacy of targeted therapy in patients with non–small cell lung cancer with gene mutation between the groups with and without cognitive disorder (CD).

Efficacy	Group		Chi-square (*df*)	*P* value
	CD (n=45)	No CD (n=42)		
PR^a^, n (%)	17 (38)	33 (79)	15.1 (2)	<.001
SD^b^, n (%)	22 (49)	8 (19)	15.1 (2)	<.001
PD^c^, n (%)	6 (13)	1 (2)	15.1 (2)	<.001
ORR^d^ (%)	37.8	78.6	15.1 (2)	<.001
DCR^e^ (%)	86.7	97.6	15.1 (2)	<.001

^a^PR: partial response.

^b^SD: stable disease.

^c^PD: progressive disease.

^d^ORR: objective response rate.

^e^DCR: disease control rate.

### Correlation Between Cognitive Disorder and Targeted Therapy

**Figure 2:**
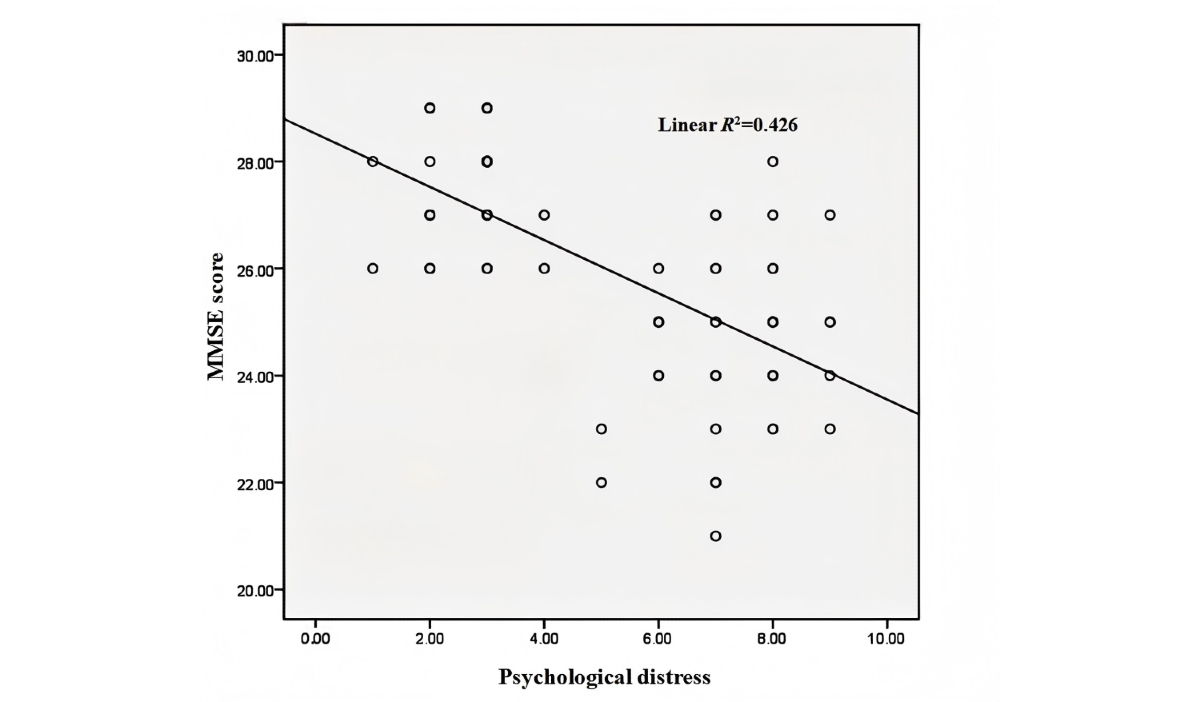
Correlation between the Mini-Mental State Examination (MMSE) score and psychological distress after targeted therapy (*R*^2^=0.426; *P*<.001).

Table 3 shows changes in the cognitive function scores of patients with NSCLC before and after targeted therapy. Before targeted therapy, the mean MMSE score of the group with no cognitive disorder was 27.93 (SD 0.75), whereas the mean MMSE score of the group with cognitive disorder was 25.51 (SD 0.63). The difference between the groups was statistically significant (*z*=−8.245; *P*<.001). After targeted therapy, the mean MMSE score of the group with no cognitive disorder was 27.48 (SD 1.06), and the mean MMSE score of the group with cognitive disorder was 24.62 (SD 1.50). The difference between the groups was statistically significant (*z*=−7.118; *P*<.001). After targeted therapy, both groups’ MMSE scores decreased, with the mean change in the group with no cognitive disorder being −0.45 (SD 1.09) and that in the group with cognitive disorder being −0.89 (SD 1.25). The group with cognitive disorder showed a more significant decrease than the group with no cognitive disorder, and the difference between the 2 groups was significant (*z*=−4.689; *P*<.001). The group with no cognitive disorder had a significant difference from before to after targeted therapy (*z*=−2.534; *P*=.01) and the cognitive disorder group also had a significant difference from before to after targeted therapy (*z*=−3.944; *P*<.001). As shown in Figure 2, there was a negative correlation between the MMSE score and the psychological distress score after targeted therapy (*R*^2^=0.426; *P*<.001).

**Table 3:** Differences in Mini-Mental State Examination (MMSE) score at different time points.

	No CD^a^ group (n=42), mean (SD)	CD group (n=45), mean (SD)	*z* score	*P* value
MMSE score at time 1^b^	27.93 (0.75)	25.51 (0.63)	−8.245	<.001
MMSE score at time 2^c^	27.48 (1.06)	24.62 (1.50)	−7.118	<.001
MMSE score at time 1 – MMSE score at time 2^d^	−0.45 (1.09)	−0.89 (1.25)	−4.689	<.001

^a^CD: cognitive disorder.

^b^Before targeted therapy.

^c^After targeted therapy.

^d^The change in MMSE score between time 1 and time 2.

### Correlation Between QoL and Cognitive Disorder

**Figure 3:**
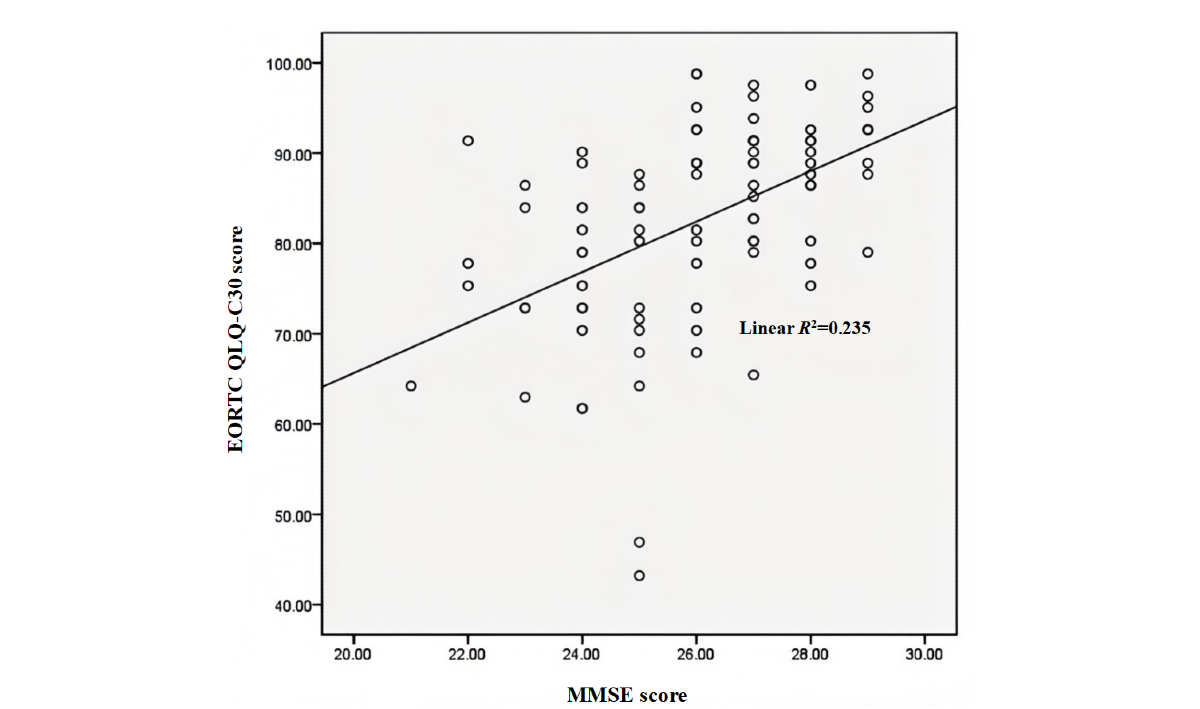
Correlation between the Mini-Mental State Examination (MMSE) score and the European Organisation for Research and Treatment of Cancer Quality of Life Questionnaire Core 30 (EORTC QLQ-C30) score after targeted therapy (*R*^2^=0.235; *P*<.001).

Before targeted therapy, the QoL score (mean 88.83, SD 5.69) of the group with no cognitive disorder was higher than that of the group with cognitive disorder (mean 80.30, SD 9.62), and there was a significant difference between the 2 groups (*t*=–5.073; *P*<.001). After targeted therapy, the QoL score (mean 90.04, SD 5.48) of the group with no cognitive disorder was higher than that of the group with cognitive disorder (mean 75.31, SD 10.40), and the difference between the 2 groups was significant (*t*=−8.337; *P*<.001). As shown in Figure 3, the MMSE score was positively correlated with QoL scores after targeted therapy (*R*^2^=0.235; *P*<.001).

### Correlation Between CRP Concentration and Cognitive Disorder

**Figure 4:**
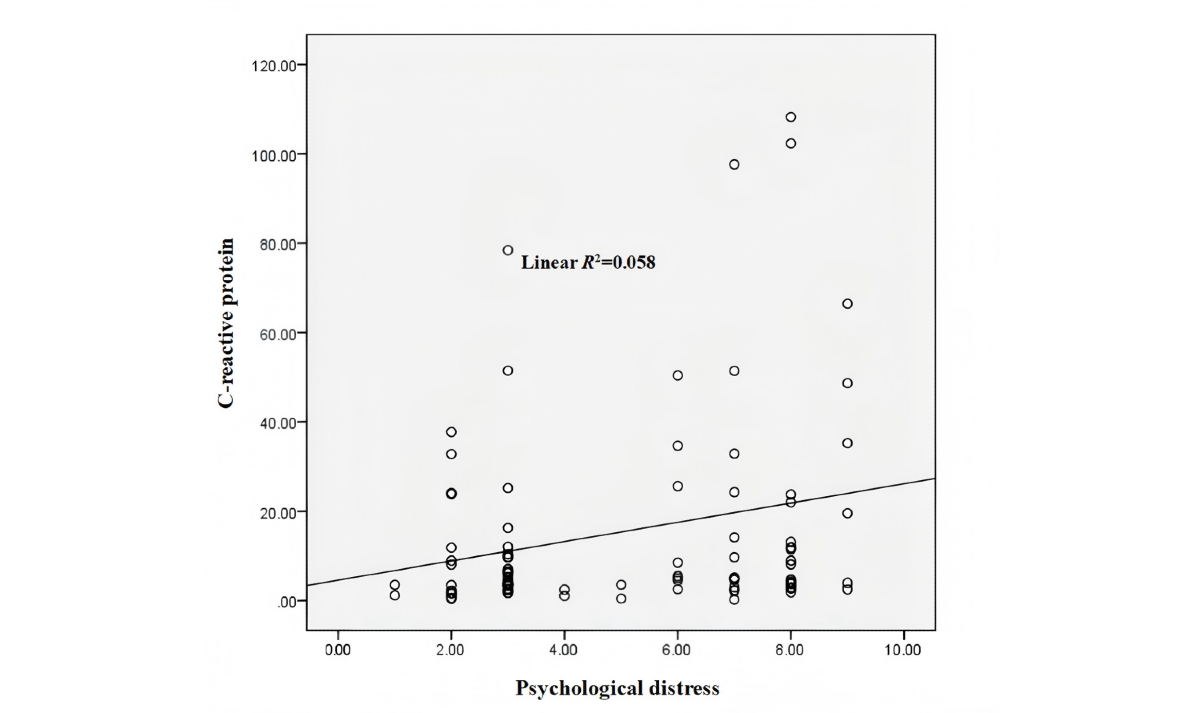
Correlation between C-reactive protein concentration and psychological distress after targeted therapy (*R*^2^=0.058; *P*=.03).

Table 4 shows that, before targeted therapy, the CRP concentration (mean 7.05, SD 10.26 mg/L) of the group with no cognitive disorder was significantly lower than that of the group with cognitive disorder (mean 16.04, SD 19.88 mg/L), and there was a significant difference between the 2 groups (*z*=−2.175; *P*=.03). After targeted therapy, the CRP concentration (mean 10.64, SD 15.37 mg/L) in the group with no cognitive disorder was also significantly lower than that of the group with cognitive disorder (mean 20.09, SD 27.37 mg/L), and there was a significant difference between the 2 groups (*z*=−1.975; *P*=.048). After targeted therapy, the changes in CRP concentration in both groups showed an increasing trend compared to before targeted therapy, and the difference between the 2 groups was significant (*z*=−3.365; *P*=.001). There is a significant difference in CRP concentration before and after targeted therapy between the group with no cognitive disorder and the group with cognitive disorder (*P*=.01 and *P*=.03, respectively). As shown in Figure 4, the psychological distress score was correlated with CRP concentration after targeted therapy (*R*^2^=0.058; *P*=.03).

**Table 4:** Differences in C-reactive protein concentration at different time points.

C-reactive protein concentration (mg/L)	No CD^a^ group (n=42), mean (SD)	CD group (n=45), mean (SD)	*z* score	*P* value
At time 1^b^	7.05 (10.26)	16.04 (19.88)	−2.175	.03
At time 2^c^	10.64 (15.37)	20.09 (27.37)	−1.975	.048
Difference between time 1 and time 2^d^	3.59 (11.12)	4.05 (18.85)	−3.365	.001

^a^CD: cognitive disorder.

^b^Before targeted therapy.

^c^After targeted therapy.

^d^The change in C-reactive protein between time 1 and time 2.

### PFS in Patients Receiving Targeted Therapy

**Figure 5:**
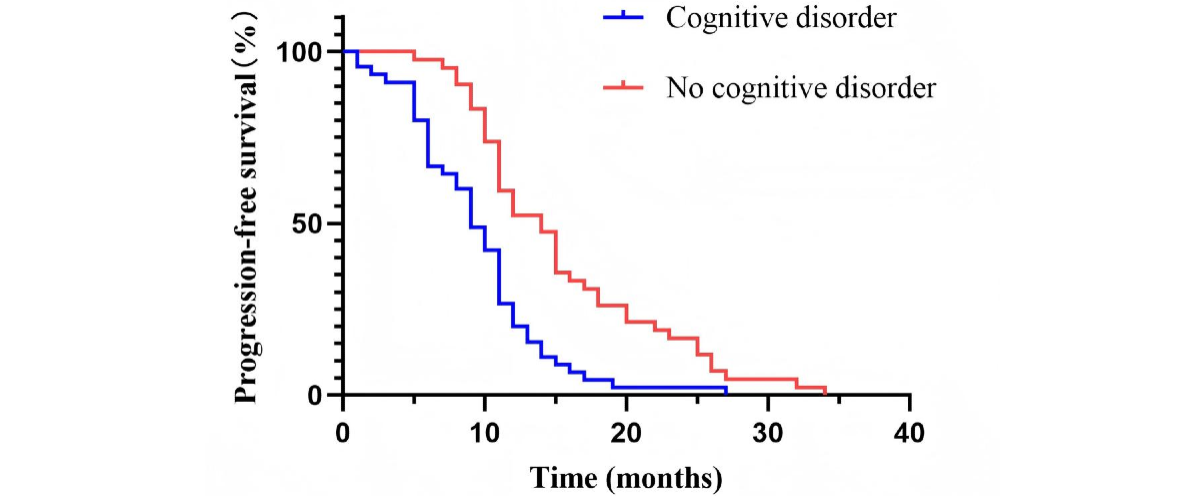
Progression-free survival in patients receiving targeted therapy for advanced non–small cell lung cancer with gene mutation.

Figure 5 shows that the PFS in patients with no cognitive disorder was significantly better than that in patients with cognitive disorder after targeted therapy (hazard ratio=0.347, 95% CI 0.209-0.578; *P*<.001). The median PFS for the group with cognitive disorder was 9 months (95% CI 7.36-10.64; IQR: 6-12 months), and the median PFS for the group with no cognitive disorder was 14 months (95% CI 11.46-16.54; IQR: 10-20 months).

### Factors Influencing PFS After Targeted Therapy

As shown in Table 5, Cox proportional hazard regression analysis revealed that sex (*P*=.67), age (*P*=.27), educational level (*P*=.85), EORTC QLQ-C30 score (*P*=.49), type of targeted drug (*P*=.56), CRP concentration (*P*=.37), and brain metastasis(*P*=*.*66) had no significant impact on PFS. However, cognitive dysfunction level was significantly associated with PFS. Cognitive disorder had a negative effect—worse cognitive function was correlated with reduced PFS (*P*=.03). Furthermore, cognitive dysfunction was identified as an independent risk factor for shorter PFS.

**Table 5:** Cox proportional hazard regression model analysis for progression-free survival in patients receiving targeted therapy.

Influence factor	Hazard ratio (95% CI)	*P* value
Cognitive function	1.946 (1.051-3.605)	.03
Sex	1.116 (0.679-1.833)	.67
Age	1.323 (0.807-2.168)	.27
Educational level	1.028 (0.765-1.382)	.85
EORTC QLQ-C30^a^ score	1.228 (0.684-2.205)	.49
Targeted drug	0.939 (0.759-1.161)	.56
Brain metastasis	0.877 (0.494-1.560)	.66
C-reactive protein concentration	1.249 (0.769-2.029)	.37

^a^EORTC QLQ-C30: European Organisation for Research and Treatment of Cancer Quality of Life Questionnaire Core 30.

## Discussion

### Principal Findings

This study suggests that cognitive disorder is associated with QoL and clinical outcomes in patients with advanced NSCLC receiving targeted therapy. The ORR was 37.8%, and the DCR was 86.7% in patients with cognitive disorder compared to 78.6% (ORR) and 19% (DCR) in patients with no cognitive disorder, demonstrating statistically significant differences in treatment efficacy between groups (*P*<.05). These results indicate superior therapeutic outcomes in patients with no cognitive disorder. Our analysis revealed a positive correlation between QoL and MMSE scores in patients with advanced NSCLC with driver gene mutations following targeted therapy (*R*^2^=0.235; *P*<.001), suggesting an association between cognitive function and posttreatment QoL. This finding aligns with those of previous studies demonstrating that cognitive decline adversely affects QoL [39-42]. Furthermore, PFS was significantly shorter in patients cognitive disorder (median 9 months) than in patients with no cognitive disorder (median 14 months), with a statistically significant between-group difference (*P*<.05).

Cancer-related cognitive disorder is not just manifested in memory loss but also in declines in language and executive functions [43]. Previous studies have confirmed that cognitive disorder is present in patients with tumors before treatment. For example, in a study of cognitive function in patients with breast cancer, compared with a healthy control group, lower cognitive function was exhibited before treatment [44,45]. In colorectal cancer cognitive function research, Cruzado et al [40] also found that 37% of patients had cognitive disorder before treatment. Similarly, Vardy et al [46] also reported in a study of colon cancer that approximately 40% of patients compared with the healthy group had cognitive disorder before treatment. In lung cancer research, 35.4% of patients with advanced lung cancer had perceptual attention disorders before treatment, and 58.4% of patients had perceptual memory disorders [29]. In this study, 52% (45/87) of patients showed cognitive disorder before treatment. As for the mechanisms of cognitive function changes in patients with cancer, Zhang et al [47] found that changes in cognitive function may be related to increased blood-brain barrier leakage. Compared to a healthy control group, patients with advanced lung cancer without brain metastasis had higher leakage rates in both temporal and whole brain lobes, primarily in visual space and executive function as well as delayed recall impairments. This could further cause brain metastasis and cognitive deterioration with the increase of blood-brain barrier leakage [47]. The research by Vannorsdall et al [48] indicates that not only does disease progression affect a patient with cancer’s perception of cognitive function, but cancer treatment also leads to deteriorating cognitive function. Increasingly, studies have found that commonly used chemotherapy in tumor treatment can cause changes in a patient’s cognitive function [49-51]. Cognitive changes are not limited to chemotherapy. There are cognitive changes even in targeted treatment. For example, previous studies have found an impact on cognitive function when vascular endothelial growth factor inhibitors are used, mainly affecting working memory and information processing speed [52]. In patients with EGFR mutations, after using EGFR tyrosine kinase inhibitor drugs, an increase in brain white matter damage and loss of gray matter volume were observed when testing using the modified Scheltens visual rating scale and voxel-based morphometry method. This reflects a possible negative impact of targeted treatment on cognitive function, which might explain the cognitive decline after targeted treatment [53]. In this study, after targeted treatment, the MMSE scores of the groups both with and without cognitive disorder decreased, with the group with cognitive disorder showing a larger decrease. Meanwhile, multivariate Cox analysis also demonstrated that cognitive disorder was an independent risk factor for PFS in patients. This step indicates that cognitive disorder impairs the therapeutic effect of targeted treatment and targeted treatment, in turn, aggravates the degree of cognitive dysfunction.

In this study, a change in the concentration of CRP was also observed. Before targeted therapy, the group without cognitive disorder had a lower concentration of CRP, with a significant difference between the 2 groups. An increasing trend in CRP levels was observed in both groups after targeted therapy. Wersching et al [54] also found a correlation between CRP levels and cognitive function, where higher CRP levels were associated with poorer executive function. This may be due to low-grade inflammation affecting the disintegration of the cerebral microstructure, hence influencing the frontal lobe pathway and corresponding executive functions and, consequently, impacting cognitive function [54]. In a large cohort study conducted by Arce Rentería et al [55] on individuals over the age of 45 years, higher CRP levels were found to be correlated with poorer memory and language fluency; CRP levels can serve as biomarkers for cognitive disorder in individuals over the age of 45 years [55]. CRP is a significant inflammatory and vascular disease biomarker. Inflammation can cause endothelial cell dysfunction, reflected in alterations in the white matter signal, which serves as a radiographic marker for cerebral vascular disease, playing a pivotal role in monitoring cognitive disorder [56,57]. Other brain regions controlling cognitive function are similarly affected by inflammation, for instance, the hippocampus. The hippocampus, located between the thalamus and medial temporal lobe and part of the limbic system, is primarily responsible for memory functions. Its volume can decrease under inflammatory conditions, closely tied to a decrease in cognitive function [58]. Hence, it can be inferred that an increase in CRP concentration is a critical indicator to predict cognitive disorder. In this study, an increasing trend in CRP was observed after targeted therapy, serving as a risk precursor for changes in cognitive function. However, no statistically significant correlation was observed between MMSE scores and fluctuations in CRP levels. The MMSE provides a composite score encompassing multiple cognitive domains (including orientation, memory, attention, language, and visuospatial function) without distinguishing specific subdomain impairments. Consequently, this global assessment metric may fail to detect subtle yet potentially clinically relevant cognitive changes associated with inflammatory processes. Future studies incorporating domain-specific neuropsychological assessments or more sensitive biomarkers of neuroinflammation may help elucidate this relationship. Targeted therapy might have promoted changes in CRP levels, resulting in changes in cognitive function.

Psychological distress is also a common psychological factor of concern in advanced lung cancer. In this study, psychological distress was positively correlated with concentrations of CRP (*R*^2^=0.058; *P*=.03) and negatively correlated with MMSE scores (*R*^2^=0.426; *P*<.001). Psychological distress is closely related to changes in cognitive function and inflammatory mediators during the onset and progression of cancer. Inflammatory cytokines are involved in the development of neuropsychiatric symptoms and depression and the risk factors associated with increased circulating inflammatory cytokines and CRP [59]. In patients with cancer as well as depression and fatigue, there are also elevated circulating inflammatory cytokines, and peripheral blood interleukin (IL)-1β, IL-6, tumor necrosis factor, and CRP levels show an increasing trend [60,61]. Among them, high plasma levels of IL-6 are related to accelerated tumor progression, high-stress states, depression, and abnormal behavior [62]. Therefore, CRP as an important indicator of inflammation is closely related to the psychological factors of patients with cancer. Similarly, clinical studies have found a correlation between psychological distress and cognitive disorder. In a large study, the higher the severity of psychological distress in young patients with cancer, the greater the probability of cognitive disorder [63]. Among patients with breast cancer, compared with those without depression, those in the depressed group scored higher on the Cognitive Error Questionnaire, indicating cognitive decline [64]. Moreover, research by Ando-Tanabe et al [65] has also confirmed that patients with greater psychological distress have higher levels of cognitive disorder. For patients with cancer during diagnosis and treatment, sufficient attention must be paid to psychological and cognitive issues, which have a certain impact on the diagnosis and treatment of tumors. Screening, consultation, and intervention involving professional oncology psychologists are necessary.

With the widespread promotion of gene testing technology in clinical applications, patients with advanced lung cancer have more treatment opportunities. Throughout the clinical treatment process, we observed changes in patients’ cognitive function. For patients with EGFR or ALK mutations, we adopted the MMSE assessment scale, EORTC QLQ-C30 scale, and DT scale to evaluate the relationship between changes in cognitive function during targeted treatment and QoL and psychological distress and cognitive function’s impact on treatment outcomes. In future treatments, patients requiring targeted therapy should routinely undergo cognitive function assessments. Adjuvant neuroprotective agents should be used to mitigate cognitive decline. Comprehensive supportive care should be implemented involving a multidisciplinary team approach with specialties such as psycho-oncology and neurology to collaboratively participate in the treatment. The EORTC QLQ-C30 should be integrated with cognitive scales to comprehensively capture treatment efficacy.

### Limitations

This study is a small-sample investigation that conducted exploratory observational research based on clinically observed phenomena. The sample size of this study is similar to that of previous clinical trials assessing cognitive function [66-68]. However, a small sample size may reduce the study’s statistical power and increase the likelihood of false-negative results. The reasons for the limited sample size are as follows. First, economic constraints played a role as some patients declined genetic testing due to the high costs, thereby losing the opportunity for targeted therapy. Second, delays in genetic test results led some patients to opt for chemotherapy before targeted therapy was available rather than receiving targeted treatment alone. Third, selection bias may have been introduced as socioeconomic factors and individual patient preferences influenced the demographic undergoing testing, potentially skewing the study sample. Fourth, the increasing number of clinical trials for lung cancer may have led some patients to participate in free clinical studies, further reducing the sample size of this trial.

### Future Directions

Moving forward, it is imperative to conduct more in-depth investigations into the precise molecular mechanisms underlying the association between targeted anticancer therapies and treatment-related cognitive dysfunction. A particularly critical area of inquiry involves elucidating the pathophysiological pathways through which molecularly targeted agents may induce or exacerbate neurocognitive impairments, potentially through blood-brain barrier disruption, neuroinflammation, or direct neuronal toxicity.

Furthermore, the potential synergistic neurotoxic effects of combination regimens incorporating both traditional chemotherapeutic agents and novel targeted therapies warrant rigorous scientific exploration. This line of investigation should use comprehensive neuropsychological assessments coupled with advanced neuroimaging and biomarker analyses to determine whether such combination therapies produce additive or multiplicative adverse effects on cognitive function. These research directions have significant clinical implications for optimizing treatment strategies while preserving patients’ QoL and neurocognitive health. In the future, we need to further investigate the molecular mechanisms linking targeted therapy and cognitive disorder. Moreover, whether chemotherapy combined with targeted therapy exacerbates cognitive disorder is also a valuable aspect requiring further exploration and research.

### Conclusions

The findings confirmed that, following targeted therapy, patients with preexisting cognitive disorder showed markedly reduced QoL metrics and inferior PFS outcomes relative to cognitively intact patients. Substantial correlation was found among psychological factors, cognitive impairment, and CRP concentration in patients with advanced lung cancer. For such patients, early screening, counseling, and effective intervention measures are crucial for enhancing clinical efficacy and QoL.

## Abbreviations

ALK: anaplastic lymphoma kinase
CRP: C-reactive protein
DCR: disease control rate
DT: Distress Thermometer
EGFR: epidermal growth factor receptor
EORTC QLQ-C30: European Organisation for Research and Treatment of Cancer Quality of Life Questionnaire Core 30
EURTAC: erlotinib versus standard chemotherapy as first-line treatment for European patients with advanced epidermal growth factor receptor mutation–positive non–small cell lung cancer
First-SIGNAL: first-line single-agent Iressa versus gemcitabine and cisplatin trial
IL: interleukin
IPASS: Iressa Pan-Asia Study
MMSE: Mini-Mental State Examination
NSCLC: non–small cell lung cancer
ORR: objective response rate
PFS: progression-free survival
QoL: quality of life

## Data Availability

The datasets generated or analyzed during this study are available from the corresponding author on reasonable request.
